# Factors associated with severe corneal endothelial damage following acute primary angle closure in Chinese subjects

**DOI:** 10.1007/s00417-023-06109-x

**Published:** 2023-05-25

**Authors:** Zhi Li, Ning Fan, Yanyan Cheng, Fei Xiang, Xiaohua Pan, Kai Cao, Ye Zhang, Qing Zhang, Shuning Li

**Affiliations:** 1 Beijing Tongren Eye Center, Beijing Tongren Hospital, Beijing Ophthalmology & Visual Science Key Lab, Capital Medical University, No. 1 Dongjiao Min Xiang, Dongcheng District, Beijing, 100730 China; 2 Shenzhen Eye Hospital, Jinan University, Shenzhen Eye Institute, Shenzhen, China; 3 Hebei Eye Hospital, Xingtai, Hebei China; 4 Beijing Institute of Ophthalmology, Capital Medical University, Beijing, China

**Keywords:** Acute angle closure, Corneal endothelium, Corneal damage, Endothelial cell density

## Abstract

**Purpose:**

To investigate the corneal endothelial damage caused by acute primary angle closure (APAC) and related risk factors for severe corneal endothelial cell damage in Chinese subjects.

**Methods:**

In this multicentre retrospective study, 160 Chinese patients (171 eyes) diagnosed with APAC were recruited. Endothelial cell density (ECD) and morphological changes short after APAC were studied. Univariate regression and multivariate regression were used to identify risk factors associated with the extent of ECD reduction, including age, gender, education level, patients’ location, systemic diseases, APAC duration (hours), highest recorded intraocular pressure (IOP), and presenting IOP. Factors associated with the probability of severe corneal damage (ECD lower than 1000/mm^2^) were analysed based on a linear function.

**Results:**

After one APAC episode, 12.28% eyes had ECD lower than 1000/mm^2^, 30.41% had ECD between 1000 and 2000/mm^2^, and 57.31% had ECD more than 2000/mm^2^. Attack duration was the only factor associated with severe endothelial damage (*p* < 0.0001). If the attack were to be subsided within 15.0 h, possibility of ECD lower than 1000/mm^2^ could be controlled under 1%.

**Conclusion:**

Shortly after the abortion of APAC, 12.28% patients experienced severe endothelial cell damage with ECD less than 1000/mm^2^. The only factor associated with severe ECD decrease was attack duration. Immediate and effective treatment is pivotal for preserving corneal endothelial function in APAC patients.


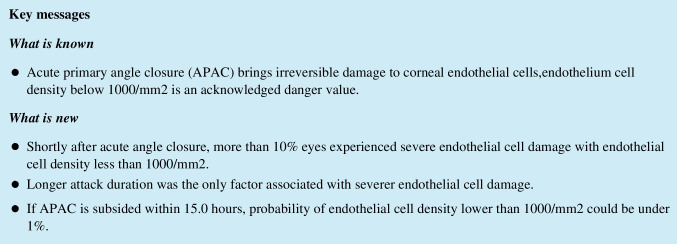


## Introduction

Acute primary angle closure (APAC) is an ophthalmic emergency where intraocular pressure (IOP) rises drastically and rapidly owning to the sudden closing of the anterior chamber angle [1]. Notably, IOP in APAC not only is imposed to the optic nerve head causing glaucomatous optic neuropathy, but also strikes the eye anteriorly, precipitating corneal oedema and loss of the endothelial cells [2–4].

Corneal endothelium is a monolayer of hexagonal cells with no proliferative capacity, executing the function of a barrier and pump, so that cornea transparency can be preserved [5]. Previous research had observed significant corneal dysfunction after APAC and reported the percentage of cell density decrease between 9.7 and 35.1% [6–12]. However, in light of the published studies, a minimal endothelial cell density (ECD) is required to perform this duty [13]. Furthermore, it is generally accepted by researchers and clinicians that the danger threshold of ECD for intraocular surgeries is 1000/mm^2^ [14–17]. Since most APAC patients in China rely on surgically aborting the attack, sustaining a proper number of ECD is essential for averting corneal decompensation postoperatively as well as during the follow-up time.

A clear correlation has been well-demonstrated between time duration of the acute attack and the extent of ECD loss. Setala et al. [6] indicated that duration of IOP elevation is a more determinant factor than its magnitude in terms of APAC-induced corneal damage. In Sihota et al.’s research [9], APAC eyes in which the attack persisted for more than 72 h had significantly lower cell counts as compared to eyes in which the attack resolved earlier. Chen et al. [11] showed that APAC duration was the only factor independently associated with ECD. However, how long exactly the attack duration will result in clinical meaningful low ECD, for example, less than 1000/mm^2^, remains uncertain.

The aim of this study is to investigate APAC-induced corneal endothelial damage and ascertain related risk factors for severe endothelial damage.

## Methods

### Study design and participants

A retrospective observational study was performed for APAC patients who sought medical aid from November 2021 to November 2022 at glaucoma clinics in 3 eye centres in China: Beijing Tongren Hospital, Shenzhen Eye Hospital, and Hebei Province Eye Hospital. Medical records of consecutive cases with previous APAC attacks were retrospectively reviewed.

Approval had been received from the institutional review board and ethics committee of Beijing Tongren Hospital (TRECKY2018-078). The study was registered with the Chinese Clinical Trial Registry (ChiCTR-ROC-17013572), complied with the Health Insurance Portability and Accountability Act regulations, and adhered to the tenets of the Declaration of Helsinki.

The patients all conform to the diagnosis of APAC [18–20] defined as follows: (1) presence of at least two of the following symptoms: ocular or periocular pain, nausea and/or vomiting, an antecedent history of intermittent blurring of vision with halos; (2) presenting IOP of > 21 mmHg (as measured by non-contact tonometry with a measuring range 0–60 mmHg; Canon TX-20, Canon Inc., Tokyo, Japan); (3) presence of at least one of three of the following signs: conjunctival injection, corneal epithelial oedema, mid-dilated unreactive pupil; (4) presence of shallow anterior chamber with slit-lamp exam and closed angle on gonioscopic exam in the eyes with APAC and a fellow unaffected eye with the presence of 180° or more of iridotrabecular contact with or without peripheral anterior synechia on gonioscopic exam.

The exclusion criteria were as follows: (1) patients presenting with secondary angle closure, such as neovascular glaucoma, uveitic glaucoma, glaucoma with severely dislocated lens, glaucoma secondary to trauma, or glaucoma after retinal laser or surgeries; (2) patients with any other primary corneal abnormalities or secondary corneal damage (including trauma and inflammation); (3) patients who were unable to cooperate during specular microscopy, which may result in poor quality imaging; (4) patients with any history of previous intraocular surgery or laser procedure, except for a single performance of laser peripheral iridotomy (LPI) and/or argon laser peripheral iridoplasty (ALPI); (5) patients with contact-wearing history; (6) patients with incomplete medical records; (7) patients with recurrent or intermittent attacks which precluded relatively accurate determination of attack duration.

### Main outcome and measures

All participants received a slit-lamp examination after APAC and gonioscopy when the corneal oedema had resolved. Instant fundus examinations were hindered in many cases because of oedematous cornea. Central ECD along with endothelial cell size (average, maximum, and minimum), standard deviation (SD), coefficient of variance (CV), and hexagonality was measured in each recruited eye using non-contact specular microscopy (TOMEY EM-4000, Tomey Corporation, Phoenix, AZ, USA). Measurements were done after IOP was controlled and the cornea restored transparency, and corneal parameters were collected before any surgical intervention. Median time from controlling of the attack to specular microscopy was 8 days (range 2 to 26 days). Moreover, endothelial cell loss (ECL) and percentage of endothelial cell loss (ECLP) were calculated. Since patients would only receive corneal examination after the attack and specular microscopy was not routine for healthy individuals, corneal measurements in advance of APAC were limited in this study. Therefore, while data of the affected eye with unilateral APAC and both eyes in patients with bilateral APAC were selected for evaluation, ECD of the unaffected fellow eye in unilateral attacks was introduced as substitute for ECD status prior to the onset of APAC. The exclusion criteria for unaffected fellow eye were as follows: history of APAC, any biomicroscopically detected corneal abnormalities, any known topical medication, any history of previous intraocular surgery or laser procedure, and contact lens wearing history. If the attack was bilateral or the fellow eye was excluded, ECD in age-stratified healthy Chinese population reported by previous research was used as replacement [21].

Information extracted from medical record systems and then used for data analysis consisted of the following: patients’ age, gender, highest education level, rural or urban location (rural location is defined as 20 km from the centre of Beijing and Shenzhen cities and 10 km from the centre of Xingtai city), systemic disease records (including high blood pressure and diabetes mellitus), attack duration, Snellen best-corrected visual acuity (BCVA), highest recorded IOP (mmHg) during the acute attack, presenting IOP (mmHg) at first medical visit, and treatment methods of final controlling of IOP.

### Statistical analysis

Statistical analyses were performed using SAS OnDemand for Academics (SAS Institute, Cary, NC, USA). Shapiro-Wilk test was performed to test for data distribution of normality. Measurements were described as mean ± SD for normal distributional data and median (P25, P75) for skew distributional data. Categorical data were described in frequencies. Differences between groups were compared using Wilcoxon test and Kruskal-Wallis test with Bonferroni correction for multiple comparison. For univariate analysis, each risk factor was tested individually for association with ECD using Wilcoxon tests for categorical variables and Spearman correlation for continuous variables. The variables with *p* < 0.2 along with other factors we were interested in were selected for multivariable analysis. This criterion is chosen based on practical experience that a variable with *p* < 0.2 provides some predictive power without adding a significant amount of variation [22]. Multivariate analysis was conducted using Poisson regression and then negative binomial regression due to over-dispersion. Beyond that, linear, exponential, logarithmic, and power functions were developed to fit the data in quest of a final predictive model, to explain the relationship between probability of severe endothelial damage and time from onset to subsidence of APAC. Function with the smallest residual sum of squares was selected to identify the critical time window with the cut-off value of 1% for ECD < 1000/mm^2^. A *p* value of < 0.05 was considered statistically significant.

## Results

### Patient characteristics

Medical records of 160 subjects (171 eyes) diagnosed with APAC at the 3 above-mentioned centres from November 2021 to November 2022 were retrieved and analysed. Demographic characteristics at enrolment are demonstrated in Table 1. Median age of the study population was 67 (59, 72) years, which mainly consisted of females (81.25%). All subjects were Chinese. Among 160 patients, 76 (47.50%) had attack in the left eye, 73 (45.63%) in the right eye, and 11 (6.87%) suffered from bilateral attack. More than half patients reported an education level of middle school or lower (53.13%), while 43.75% received education higher than middle school. Educational information for the rest 3.12% failed to be surveyed. In terms of location, the majority resided in rural settings (65.00%) and the remaining 35.00% were urban residents. All 171 APAC-affected eyes received treatment, which composed topical and/or systemic antiglaucoma medications alone as final IOP-controlling method (5.85%), medication combined with laser therapy of LPI and/or ALPI (7.02%), and eventual surgical treatment (87.13%). Of 160 patients, 26.88% had systemic disease of high blood pressure and 21.25% were diabetic.

**Table 1:** Demographic characteristics of patients with APAC (*N* = 160)

Mean age, years (P25, P75)	**67 (59, 72)**
Gender, *n* (%)	
Male	30 (18.75%)
Female	130 (81.25%)
Affected eye, *n* (%)	
Left	76 (47.50%)
Right	73 (45.63%)
Bilateral	11 (6.87%)
Education level, *n* (%)	
Middle school or lower	85 (53.13%)
Higher than middle school	70 (43.75%)
No information	5 (3.12%)
Location, *n* (%)	
Rural	104 (65.00%)
Urban	56 (35.00%)
Treatment methods of final controlling IOP, *n* (%) (*N* = 171 eyes)	
Medication, *n* (%)	10 (5.85%)
Laser (including LPI and ALPI), *n* (%)	12 (7.02%)
Surgery, *n* (%)	149 (87.13%)
Systemic diseases, *n* (%)	
High blood pressure	43 (26.88%)
Diabetes mellitus	34 (21.25%)

*IOP*, intraocular pressure; *LPI*, laser peripheral iridotomy; *ALPI*, argon laser peripheral iridoplasty

Ocular and corneal endothelial characteristics at enrolment are demonstrated in Table 2. The median duration was 24 h, ranging from 1 to 196 h. Five in 171 (2.92%) APAC eyes had vision of only light perception, while 2 (1.17%) had no light perception at all. The median for highest recorded IOP during attack was 50 mmHg, and median for presenting IOP at first medical visit was 29.90 mmHg. After one single episode of APAC, median ECD was 2158.60/mm^2^, witnessing a median reduction of 534.00/mm^2^ (19.43%). As for the fellow eye, median ECD was 2739.00/mm^2^. ECD in the affected eyes was significantly lower compared to the unaffected fellow eyes (*p* < 0.0001, Wilcoxon signed rank test).

**Table 2:** Ocular and corneal endothelial characteristics of eyes with APAC (*N* = 171)

Attack duration, hours (P25, P75)	**24 (12, 72)**
LogMAR VA (P25, P75)	2.303 (0.916, 4.269)
Light perception, *n* (%)	5 (2.92%)
No light perception, *n* (%)	2 (1.17%)
Maximum IOP, mmHg (P25, P75)	50.00 (41.00, 60.00)
Presenting IOP, mmHg (P25, P75)	29.90 (13.40, 51.00)
Cell density, /mm^2^ (P25, P75)	2158.60 (1536.40, 2558.00)
Average cell size, μm^2^ (P25, P75)	446.80 (384.00, 650.00)
SD, μm^2^ (P25, P75)	1190.40 (141.10, 203.20)
CV (SD)	38.50 (33.00, 45.30)
Hexagonality, % (P25, P75)	43.0 (30.0, 57.0)
Fellow eye cell density, /mm^2^ (P25, P75)	2739.00 (2589.00, 2803.30)
ECL, /mm^2^ (P25, P75)	534.00 (40.50, 1135.40)
ECLP, % (P25, P75)	19.43 (1.58, 41.53)

*VA*, visual acuity; *SD*, standard deviation; *CV*, coefficient of variance; *ECL*, endothelial cell loss; *ECLP*, percentage of endothelial cell loss

### Comparison between subgroups

APAC eyes were then divided into subgroups according to the severity of ECD impairment, with ECD more than 2000/mm^2^ defined as group 1 (98 eyes, 57.31%), ECD ranging from 1000 to 2000/mm^2^ as group 2 (52 eyes, 30.41%), and ECD less than 1000/mm^2^ as group 3 (21 eyes, 12.28%). Demographic and endothelial parameters of each group are shown in Table 3. There were no differences observed in terms of age, gender, education level, location, or systemic diseases among 3 groups (all *p* > 0.05). However, eyes with ECD lower than 1000/mm^2^ did have larger proportions of lower education level than other groups. Eyes in group 3 had higher maximum IOP as well as presenting IOP than eyes in group 1 and group 2, though the differences were insignificant (both *p* > 0.05). The statistical insignificance remained after multiple comparison (both *p* > 0.0167). The median attack duration in group 3 was 120 h (25th percentile was 72 h and 75th percentile of 168 h), which is significantly longer than the other 2 groups (both *p* < 0.0001). As for corneal endothelial measurements, average cell size and minimum cell size differed significantly between 3 groups (both *p* < 0.0001), and the differences remained significant after multiple comparison (all *p* < 0.0167). Compared with group 1, eyes in group 2 had higher SD, CV, larger maximum cell size, and lower hexagonality (*p* < 0.0001, = 0.0010, < 0.0001, < 0.0001, and < 0.0001 respectively). Yet, no such differences were found between group 2 and group 3 (all *p* > 0.0167). In addition, ECL and ECLP went up as ECD decreased, showing significant differences in multiple comparison.

**Table 3:** Comparison between different severity of baseline corneal endothelial damage

Severity of corneal damage	Group 1 (*n* = 98)	Group 2 (*n* = 52)	Group 3 (*n* = 21)	*P*	*P*^a^	*P*^b^	*P*^c^
Mean age, years	65.42 ± 8.75	67.13 ± 9.47	67.43 ± 9.81	0.4392	> 0.05	> 0.05	> 0.05
Female, *n* (%)	77 (78.57)	43 (82.69)	18 (85.71)	0.6850	NA	NA	NA
Education of middle school or lower, *n* (%)	50 (51.55)	29 (59.18)	13 (65.00)	0.4464	NA	NA	NA
Rural location, *n* (%)	66 (67.35)	32 (61.54)	14 (66.67)	0.7704	NA	NA	NA
High blood pressure, *n* (%)	28 (28.57)	14 (26.96)	3 (14.29)	0.3996	NA	NA	NA
Diabetes mellitus, *n* (%)	24 (24.49)	8 (15.38)	2 (9.52)	0.1844	NA	NA	NA
LogMAR VA	2.30 (0.92, 4.27)	2.57 (0.92, 4.27)	3.11 (1.30, 4.27)	0.2450	0.3678	0.1140	0.3852
Attack duration, hours	20 (4, 24)	72 (48, 96)	120 (72, 168)	< 0.0001*	< 0.0001^#^	< 0.0001^#^	0.0135^#^
Maximum IOP, mmHg	50 (40, 58)	50 (43, 57)	53 (42, 60)	0.5329	0.7337	0.2646	0.4195
Presenting IOP, mmHg	37.85 (15, 51)	23.5 (14.25, 44.5)	40 (16, 60)	0.0704	0.0474	0.3861	0.0776
Average cell size, μm^2^	396.00 (362.40, 431.00)	641.20 (562.05, 787.95)	1172.00 (1098.40, 1390.00)	< 0.0001*	< 0.0001^#^	< 0.0001^#^	< 0.0001^#^
SD, μm^2^	148.75 (122.00, 185.30)	274.00 (224.50, 386.50)	483.00 (234.00, 678.20)	< 0.0001*	< 0.0001^#^	< 0.0001^#^	0.0325
CV	37.85 (33.00, 43.60)	43.85 (36.15, 52.70)	35.00 (21.00, 50.70)	0.0044*	0.0010^#^	0.6305	0.0703
MAX, μm^2^	892.95 (792.90, 1020.70)	1384.80 (1161.00, 1970.00)	2204.60 (1470.00, 2818.00)	< 0.0001*	< 0.0001^#^	< 0.0001^#^	0.0206
MINI, μm^2^	130.40 (101.80, 163.30)	195.80 (81.20, 305.45)	483.20 (331.10, 758.90)	0.0069*	0.0003^#^	0.0001^#^	0.0018^#^
Hexagonality, %	52.0 (38, 62.5)	33.0 (25, 50)	27.5 (0, 42.5)	< 0.0001*	< 0.0001^#^	< 0.0001^#^	0.0888
ECL, /mm^2^	118.89 ± 353.27	1034.97 ± 426.56	1948.56 ± 593.62	< 0.0001*	< 0.05*	< 0.05*	< 0.05*
ECLP, %	4.04 (− 4.97, 12.40)	40.46 (29.45, 48.93)	73.93 (65.59, 76.95)	< 0.0001*	< 0.0001^#^	< 0.0001^#^	< 0.0001^#^

*SD*, standard deviation; *CV*, coefficient of variance; *MAX*, maximum cell size; *MINI*, minimum cell size; *ECL*, endothelial cell loss; *ECLP*, percentage of endothelial cell loss; *NA*, not analysed

Data were expressed as mean ± standard deviation for normal distributional data and median (P25, P75) for skew distributional data

^*^p < 0.05 considered statistically significant

^#^*p* < 0.0167 considered statistically significant after Bonferroni correction following Kruskal-Wallis test

^a^Comparison between mild and moderate damage groups

^b^Comparison between mild and severe damage groups

^c^Comparison between moderate and severe damage groups

### Risk factors for ECD reduction

**Fig. 1:**
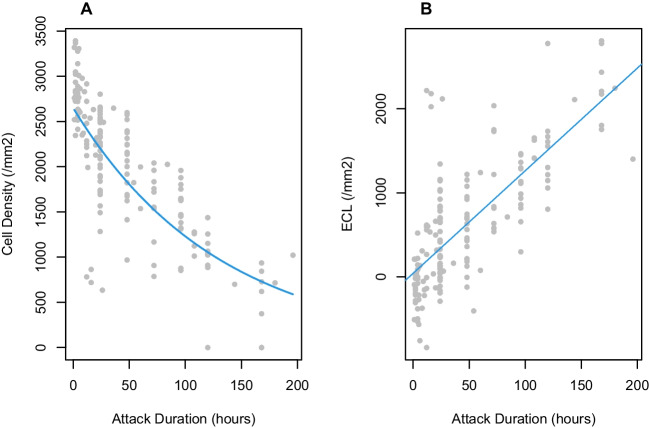
**A** Relationship between attack duration and endothelial cell density (ECD). **B** Relationship between attack duration and endothelial cell loss (ECL)

Results of univariate and multivariate analyses are shown in Table 4. In univariate analysis, among selected variables of age, gender, education level, location, systemic diseases, attack duration, highest IOP, and presenting IOP, only attack duration was found to be associated with more advanced corneal endothelial damage (*p* < 0.0001, *r* = − 0.74119). Still, we analysed maximum IOP and presenting IOP in multivariate regression along with attack duration, despite insignificant results in univariate analysis (*p* = 0.6806 and 0.3313 respectively). Attack duration remained to be the only significant variable with negative correlation with ECD after APAC (*p* < 0.0001, *β* = − 0.0081). The relationship between ECD and attack duration is demonstrated in Fig. 1 A. Beyond that, higher ECL and ECLP had shown satisfying correlation with lower ECD. We were also able to identify significant positive correlation between attack duration and ECL (*p* < 0.0001, *r* = 0.74838) (Fig. 1 B), and ECLP (*p* < 0.0001, *r* = 0.75902).

**Table 4:** Risk factors associated with corneal endothelial damage after APAC: univariate and multivariate regression analyses

Variables	Univariate regression			Multivariate regression		
	Statistical methods	*P* value	*r*	*P* value	*β*	95% CI (upper, lower)
Age	Spearman correlation	0.0772	− 0.13551	NA		
Gender	Wilcoxon test	0.7856	NA	NA		
Education level	Wilcoxon test	0.2844	NA	NA		
Location	Wilcoxon test	0.7002	NA	NA		
HBP	Wilcoxon test	0.7218	NA	NA		
DM	Wilcoxon test	0.1175	NA	NA		
Attack duration	Spearman correlation	< 0.0001*	− 0.74119	< 0.0001*	− 0.0081	(− 0.0096, − 0.0066)
Maximum IOP	Spearman correlation	0.6806	− 0.03171	0.7034	− 0.0014	(− 0.0087, 0.0058)
Presenting IOP	Spearman correlation	0.3313	0.07474	0.9718	− 0.0001	(− 0.0040, 0.0039)
ECL	Spearman correlation	< 0.0001*	− 0.89444	NA		
ECLP	Spearman correlation	< 0.0001*	− 0.90941	NA		

*ECL*, endothelial cell loss; *ECLP*, percentage of endothelial cell loss; *NA*, not analysed; *HBP*, high blood pressure; *DM*, diabetes mellitus

^*^p < 0.05 considered statistically significant

In addition, we investigated the relationship between ECD and corneal morphological parameters, as well as the relationship between morphological parameters and attack duration. There were negative correlations between ECD and SD (*p* < 0.0001, *r* = − 0.74442), CV (*p* = 0.0193, *r* = − 0.17883), average cell size (*p* < 0.0001, *r* = − 0.88141), maximum cell size (*p* < 0.0001, *r* = − 0.74107), minimum cell size (*p* < 0.0001, *r* = − 0.45188), and a positive correlation between ECD and hexagonality of the cells (*p* < 0.0001, *r* = 0.49451). Regarding attack duration, longer attack duration was associated with larger SD, CV, average, maximum and minimum cell size (all *p* < 0.0001, *r* = 0.57130, 0.10901, 0.70811, 0.57909, 0.36664 respectively), and lower hexagonality (*p* < 0.0001, *r* = − 0.40490).

### Critical time window to prevent severe endothelial damage

Based on the model described above, we further explored the relationship between probability of ECD < 1000/mm^2^ and attack duration, regardless of any other studied variables. Linear, exponential, logarithmic, and power functions were developed to fit the data in quest of a final predictive model. Linear function outstood with optimal fit (smallest residual sum of squares). The regression model is as follow: probability of ECD lower than 1000/mm^2^ = 0.00095663 + 0.00060521 × (attack duration). When the cut-off of probability is defined as ≤ 1%, the time window is 15.0 h to avert severe corneal damage.

## Discussion

APAC, as an urgent subtype of PACG, disproportionally burdens patients in Asia more than in any other regions [23–25]. When the remarkably sharp IOP elevation strikes (IOP level can be over 50 mmHg), cloudy cornea is often observed, for that the impaired endothelial layer could no longer exert its function of dehydrating the cornea [5]. Opaque cornea causes massive visual quality reduction for patients, along with diagnosis and treatment difficulties for clinicians: prohibited direct visualization of anterior segment and optic disc, performance of timely laser and surgical intervention, for instance. As the destructed cells compensatorily enlarge and migrate rather than regenerate [26], once endothelial damage exceeds a certain limit, corneal decompensation may develop, leading to potential corneal transplantation. Thus, identifying risk factors associated with severe endothelial damage to avoid corneal adverse events after APAC is of paramount significance.

Our study discussed endothelial damage short after APAC, both numerically and morphologically. Through subgroup comparisons, evident pleomorphism and polymegathism changes are observed, as group 2 and group 3 had higher SD, CV, larger maximum, and lower hexagonality than group 1. Two facts were found based on our analysis. One is that group 1 had endothelial cells with not only almost normal numbers, but also satisfying morphology after controlling the attack. The other is that corneal endothelial cells in group 2 were evidently damaged despite an ECD more than 1000/mm^2^. For group 3, however, the pleomorphism and polymegathism changes only displayed significance comparing to group 1 but not group 2.

In fact, eyes with ECD < 400–1000/mm^2^ has been proposed to be more susceptible to corneal decompensation during follow-up in previous research [14–16]. More importantly, individuals with ECD less than 1000/mm^2^ are believed to suffer from increased risk of corneal failure following intraocular surgeries. Although several studies had investigated and revealed the effect of acute angle closure glaucoma on endothelial cell loss [6, 8, 10, 11], our study identified a surprisingly large proportion (12.28%) of APAC eyes with ECD lower than 1000/mm^2^. It should be mentioned that most APAC patients in China (87.1% in our study sample) almost inevitably demand surgery for successful controlling of IOP. So, a proportion over 10% is concerning, as resultant severe endothelial damage predisposes these patients to underlying risk of cornea-related visual disabilities later. To this end, exploring the risk factors for severely low ECD shortly after APAC is meaningful for preventing corneal complications.

Among possible risk factors of age, IOP, education level, residential location, or systemic diseases, none rendered severer corneal damage. Attack duration was the only factor associated with APAC-induced endothelial loss. This result is consistent with most studies [9, 11]. Of note, we were able to further ascertain that APAC duration was significantly associated with all endothelial morphological parameters. The longer duration, the more remarkable pleomorphism and polymegathism damages, which to some extent reflected abnormal endothelial function in APAC with delayed management too.

Based on this, we built a model discussing how long the attack duration could result in severe endothelial damage. Our results indicated that the time window was 15.0 h, within which probability of ECD < 1000/mm^2^ can be extensively averted (under 1%). Consequently, long-term corneal safety may be reserved.

Still, there are some limitations to our study. To start with, estimation of APAC duration was based on patients’ self-report, which might be inaccurate and inevitably led to recall bias. Secondly, due to the retrospective design, we could not control for potential confounding effect by measurements performed by different technicians among 3 centres. The retrospective nature of our study had also prohibited corneal evaluation antecedent to the attack. Therefore, we adopted ECD in the unaffected fellow eye and in age-stratified healthy Chinese population. Lastly was the absence of follow-up. Strictly designed prospective studies are needed to reiterate our conclusion and to further observe long-term related adverse events too [17, 27].

To sum up, this study identified a 12.28% of eyes with ECD < 1000/mm^2^ after one episode of APAC in Chinese and found that attack duration was the only factor associated with severe endothelial damage. Timely treatment is key for minimizing corneal damage, as shorter attack guarantees less ECD loss and better endothelial morphology.
